# Experimental investigation of mechanical and optimization of machining parameters of nanoclay-filled glass reinforced plastic composite

**DOI:** 10.1038/s41598-026-55324-z

**Published:** 2026-06-01

**Authors:** Umeshchandra Jadhao, Nitin Ambhore, Vishal Naranje, Amol Dhumal

**Affiliations:** 1 Department of Mechanical Engineering, Vishwakarma Institute of Information Technology, Savitribai Phule Pune University, Pune, 411048 India; 2 School of Engineering, Architecture and Interior Design, Amity University Dubai, Dubai, 345019 UAE; 3 Vishwakarma Institute of Technology, Savitribai Phule Pune University, Pune, 411037 India

**Keywords:** Nanoclay, GFRP composites, Mechanical properties, Response surface methodology, Engineering, Materials science, Nanoscience and technology

## Abstract

In this study, an experiment has been conducted on the mechanical properties and machinability of nanoclay-filled glass fiber reinforced plastic composites. GFRP composites with 0, 1, 3, and 5 wt% nanoclay contents have been prepared and tested for density, tensile, and flexural properties based on ASTM procedures. Results show improved tensile and flexural strengths and moduli upon adding nanoclay, with the best results at 3 wt% nanoclay. The machinability experiments were carried out using CNC milling, and the influence of nanoclay reinforcement, spindle speed, feed rate, and cutting depth on the surface roughness (Ra) and material removal rate (MRR) was investigated using response surface methodology based on Box–Behnken design. The results of ANOVA confirmed that the proposed models were statistically significant. The nanoclay reinforcement, spindle speed, feed rate, and cutting depth were found to have a major influence on surface roughness and MRR, respectively. The multi-response optimization procedure revealed that the optimal machined layer at moderate nanoclay and spindle speed could minimize the surface roughness while maximizing the productivity. This paper offers conclusive evidence that the controlled nanoclay reinforcement improves the mechanical and machinability properties of GFRP composite materials.

## Introduction

In recent years, there has been an increase in the use of composite materials based on polymeric matrices in various engineering applications owing to their advantageous strength-weight ratio, anticorrosion properties, and design flexibility. Among these, fiber-reinforced polymeric composites have been adopted in the automotive, aerospace, and naval industries, apart from others. The traditional polymeric composites have a few limitations, such as the lack of thermal stability, low barrier properties, and diminished resistance to the propagation of cracks. To overcome these current limitations, the use of nanofillers in polymeric composites has proved to be a successful tool in modifying these composites^1,2^.

Nanoclay, a category of layered silicates with a nanometer range of platelet dimensions, has also drawn considerable attention as a potential nanofiller for polymer nanocomposites. Among different types of nanoclay, montmorillonite (MMT) has gained considerable attention owing to its higher aspect ratio, high surface area, and excellent polymer-matrix interaction^3,4^. Nanoclay with a very low fraction (usually 1–5%) of weight has also been demonstrated to significantly enhance tensile strength, bending strength, and tensile and bending moduli, along with thermal, flame, and barrier properties^5–7^.

The reinforcing efficiency of nanoclay originates mainly from its peculiar layered structure and high surface-to-volume ratio, which ensures effective stress transfer between the matrix and the filler. According to the degree of dispersion, composites based on nanoclay can have an intercalated or exfoliated morphology; indeed, exfoliated structures generally assure better property enhancement compared with intercalated structures^8,9^. However, homogeneous dispersion is still a crucial challenge because the agglomeration of nanoclay platelets at higher loadings induces mechanical performance deterioration due to the occurrence of points of stress concentration^10^.

The addition of nanoclay in GFRP composites was reported to enhance fiber-matrix adhesion, reduce the possibility of microcrack initiation, and allow for more effective load transfer^11,12^. These improvements make nanoclay-filled GFRP composites particularly attractive for high-performance structural applications. In addition, the incorporation of nanoclay has been associated with enhanced thermal resistance and reduced flammability, which are critical requirements in many aerospace and construction industries^13,14^.

However, besides the mechanical and thermal properties, nanoclay also has an effect on the machining properties and ease of machining of polymer-based composites. This is because the addition of nanoclay to any material results in the production of hard particles that can affect machining properties such as cutting force and surface finish during machining operations such as milling and drilling^15–18^. As such, it is imperative to grasp the effects of nanoclay content on material properties and machining properties. In this regard, this present work is specifically dedicated to the functional aspect of nanoclay as a nanofiller in polymers as well as glass fiber-based composites in terms of its effect on their mechanical properties, microstructure, and challenges associated with their performance. It is important to gain an in-depth insight into the interactions of nanoclay as a nanofiller in a matrix.

Although many studies have been conducted separately on either the mechanical behavior or machining properties of nanoclay/GFRP composite materials, a lack of studies that consider nanoclay content as an independent machining process parameter and conduct joint optimization analysis on surface finish and production output exists. This gap is addressed in the current research. The purpose of the current research is as follows: (1) to study the density, tensile strength, and flexural behavior of nanoclay/GFRP composite materials under 0, 1, 3, and 5 wt% loading of nanoclay content; (2) to analyze the effect of nanoclay content, spindle speed, feed rate, and depth of cut on surface roughness (Ra) and material removal rate (MRR) during CNC milling operation; (3) to build statistically reliable RSM models for Ra and MRR based on Box–Behnken design; and (4) to optimize machining parameters by applying multiple response desirability function. The outcomes of the present study can be implemented directly in aerospace structures, auto-body parts, ship hulls, and wind turbines.

## Materials and methods

The introduction of nanoclays to glass-reinforced polymer (GRP) composites profoundly affects their mechanical performance through the alteration of the microstructure, enhancement of interfacial bonding, and reinforcement of stress-transfer mechanisms in the composite^19,20^. Nanoclays, especially organo-modified montmorillonite (MMT), are composed of platelets of layered silicate with a high aspect ratio and considerable surface area that enable efficient interaction with the polymer matrix once well dispersed.

### Density test

**Fig. 1:**
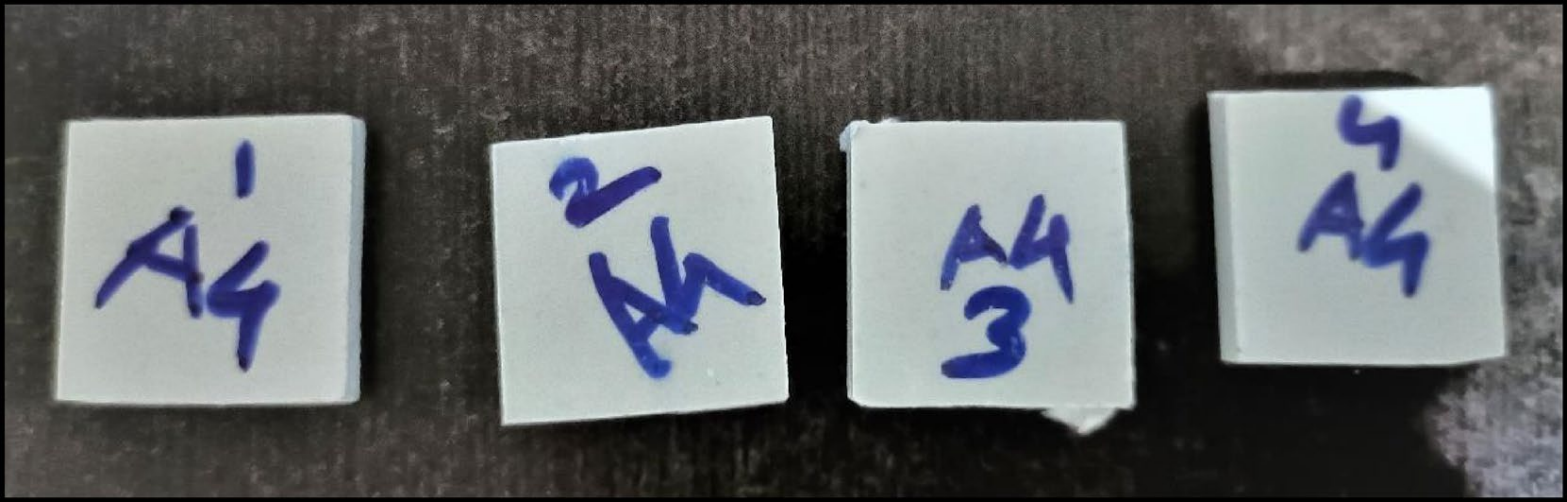
Density test specimens.

This test was conducted to determine variations in material density due to nanoclay content. All specimens were subjected to this test according to ASTM D792 as illustrated in Fig. 1. The determination of the density of solid plastics by the displacement method is described in ASTM D792. Specific Gravity Determiner (Model: ME204/A04) manufactured by Mettler Toledo was used in this test. It had a weighing capacity of 0.1–220 g and could measure up to 0.0001 g. The testing fluid was distilled water maintained at 27 °C, having a density of 1000 kg/m^3^. The specific gravity of each specimen was measured as the ratio of its weight in air to the loss in weight in water (Table 1).

**Table 1:** Specific gravity.

Batch/Specimen	Nanoclay 0 wt%	Nanoclay 1 wt%	Nanoclay 3 wt%	Nanoclay 5 wt%
Specimen No. 1	1.826	1.852	1.858	1.844
Specimen No. 2	1.844	1.856	1.869	1.842
Specimen No. 3	1.83	1.853	1.861	1.859
Specimen No. 4	1.845	1.852	1.842	1.832
Average	1.83625	1.85325	1.8575	1.84425
Average change w.r.t 0 wt%	–	0.93%	1.16%	0.44%

### Tensile and flexural properties

The tensile test was conducted to evaluate the ability of the material to withstand tensile loading and to determine its elastic behavior. Tensile testing was performed in accordance with ASTM D638 and ASTM D3039 standards, where ASTM D638 is commonly used for plastic materials, while ASTM D3039 is specifically recommended for fiber-reinforced composite materials and employs a rectangular plate-type specimen. In the present study, ASTM D3039 was adopted for composite testing. The experiments were conducted using a computerized Universal Testing Machine (UTM) manufactured by Kalpak Instruments and Controls, having a maximum load capacity of 100 kN, a load resolution of 0.01%, and a load accuracy of ± 0.5%. A 50 kN load cell was used for the tests, and the crosshead speed was maintained at 2 mm/min as specified by ASTM D3039. The tensile strength (σ_tensile_) of the specimens was calculated using the relation σ_tensile_ = P/A, where *P* is the maximum applied load, and *A* is the original cross-sectional area of the specimen.

**Fig. 2:**
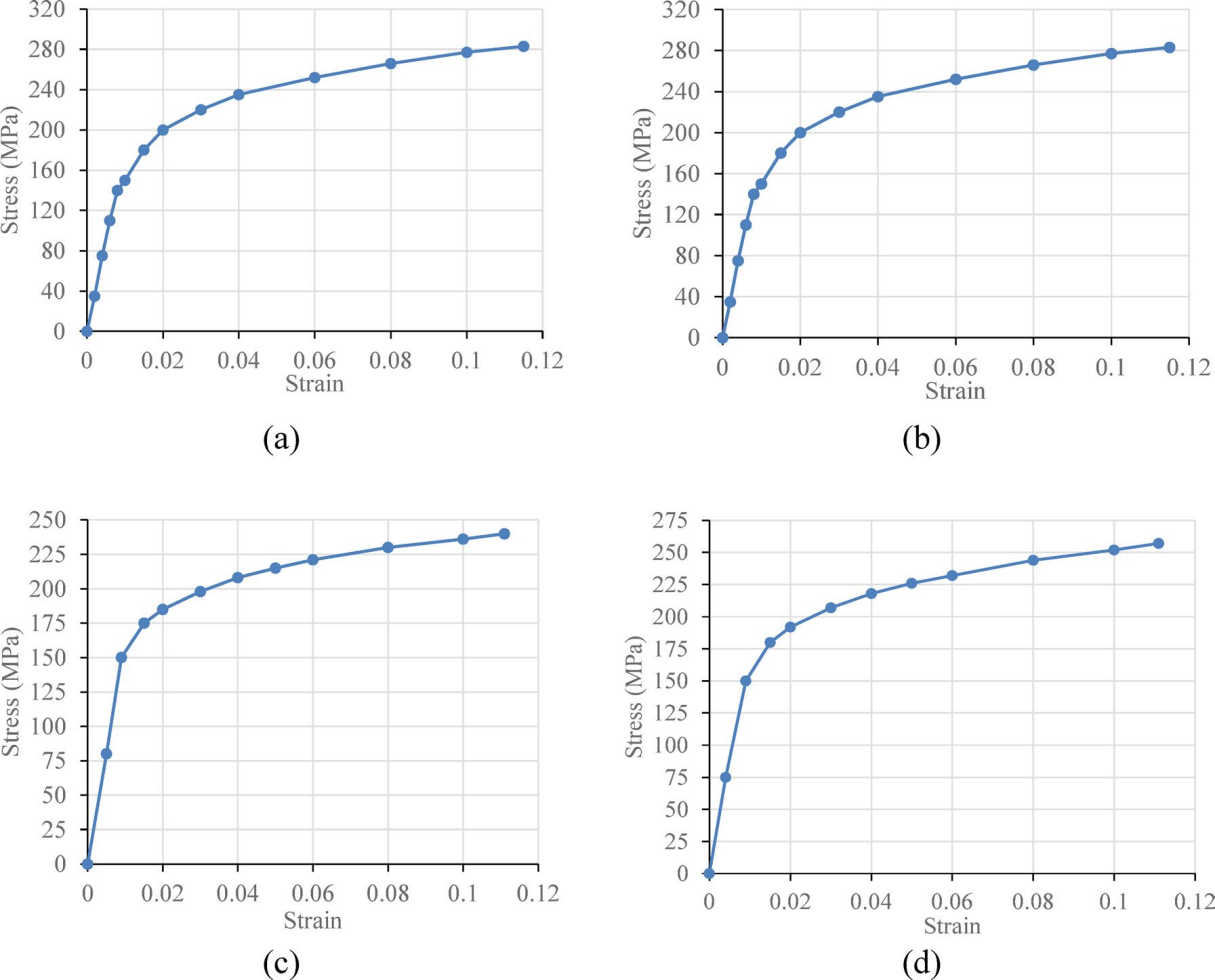
Tensile stress–strain behaviour of nanoclay-filled GRP composites at (**a**) 0 wt%, (**b**) 1 wt%, (**c**) 3 wt%, and (**d**) 5 wt% nanoclay content.

Following Table 2, consisting of the experimental findings of ultimate tensile strength and tensile modulus measured in accordance with ASTM D3039 test standards. Furthermore, the corresponding graphs of stress vs. strain curve for the specimens are also depicted in Fig. 2a–d for varied weight percentages of nanoclay. From these graphs, it can be vividly noted that, in addition to the treatment of these graphs, which reveals data regarding elastic deformation, yielding, and breaking of the specimen samples, an optimal improvement in tensile strength and tensile modulus is caused due to the presence of nanoclay. On exceeding this optimal limit, a slight decrement in the values of tensile strength and tensile modulus is also noted, due to which lesser matrix continuity is observed. Furthermore, it can also be noted that, in addition to offering superior tensile strength values, these specimens also possess high tensile modulus values.

**Table 2:** Tensile test results of nanoclay-filled GFRP composites.

Nanoclay content (wt%)	Ultimate tensile strength (MPa)	Tensile modulus (GPa)
0	240	20.8
1	257	22.5
3	283	23.1
5	252	23

Figure 2a is the stress–strain curve for neat GRP (0 wt% nanoclay). In this case, there is only linear elastic behavior, and then suddenly, the graph drops off, meaning the brittleness of this material. Due to the lack of nanoclay reinforcement in GRP, it has very low resistance against cracking. As can be seen from Table 2, the tensile strength and tensile modulus of neat GRP are 240 MPa and 20.8 GPa, respectively. These are considered low figures because of inefficient stress transfer between the polymer matrix and the glass fibers, and the presence of cracks at very early stages. Figure 2b is the stress–strain graph of 1 wt% nanoclay-reinforced GRP. Here, it can be noted that the stiffness has improved since there is a steep slope in the elastic region. As a result, the tensile strength increases to 257 MPa because of the uniform distribution of nanoclay platelets in the polymer matrix, preventing the sliding of polymer chains, thus improving the interfacial bonding. Moreover, nanoclay behaves as a microcrack bridging substance and inhibits crack formation. As can be noted in Fig. 2c, the sample containing 3 wt% nanoclay demonstrates excellent tensile strength and modulus of 283 MPa and 23.1 GPa, respectively. This major development can be attributed to the good dispersion of the nanoclay, which ensures effective transfer of loads from the matrix to the glass fiber. Furthermore, the plate-like structure of the nanoclay makes possible mechanisms like crack deflection and crack pinning. But the effect shown by Fig. 2d clearly demonstrates a decrease in tensile strength to 252 MPa on increasing the percentage of nanoclay to 5 wt%, despite maintaining a high value for the tensile modulus. The major cause behind the loss of tensile strength is the agglomeration of nanoclay.

The results for the experimental findings are compiled in Table 3. For the sample with 0 wt% nanoclay, the maximum load was 950 N, with a flexural strength and flexural modulus of 420 MPa and 18.5 GPa, respectively. The addition of 1 wt% nanoclay, however, increased the maximum load to 1030 N, with an enhanced flexural strength and modulus of 455 MPa and 20.0 GPa, respectively. Moreover, a remarkable enhancement was experienced with the addition of 3 wt% nanoclay, with a maximum load that reached 1180 N, coupled with the highest recorded flexural strength and modulus of 525 MPa and 23.5 GPa, respectively. This clearly established optimal reinforcement and efficient interfacial stress transfer between the matrix and nanoclay materials. However, with the addition of 5 wt% nanoclay, the maximum load reduced to 1050 N, with a consequent decrease in both flexural strength and modulus to 470 MPa and 21.5 GPa, respectively. This could be related to aggregation effects for the nanoclay with reduced dispersion efficiency. Based on the data compiled in Table 3, moderate amounts of nanoclay addition strikingly enhanced the flexural response, with 3 wt% nanoclay exhibiting optimal results for these mechanical properties.

**Table 3:** Flexural test results of nanoclay-filled GFRP composites.

Nanoclay content (wt%)	Maximum load (N)	Flexural strength (MPa)	Flexural modulus (GPa)
0%	950	420	18.5
1%	1030	455	20.0
3%	1180	525	23.5
5%	1050	470	21.5

## Machinability characteristics of nanoclay-filled GFRP composites

In optimization experiments, designing experiments plays an important part in improving consistency in results and reliability at a significantly reduced number of experiment trials without affecting accuracy. Response surface methodology is one of the most efficient statistical modeling and analysis techniques for controllable input variables of a system that affect the response of the system, such as surface roughness. Delamination during the machining of fiber-dominated composites is one of the most predominant machining flaws, seriously affecting surface finish and structural integrity. Inclusion of interlaminate bonding is helpful in improving delamination characteristics of composites. In this experimental investigation, nanoclay particles were mixed into the composite to improve interlaminate bonding characteristics during machining. To understand the importance of nanoclay in machining characteristics, the nanoclay weight percentage was varied in terms of the input variable. Alongside machining variables, this variable was also designated as an independent variable in designing experiments. Table 4 contains details of the independent variables chosen for designing experiments. Parameters and parameter levels for the experiment were selected from previous trial experiments, limitations of machine tooling, and information available in the literature on the machining of GFRP composite materials. The ranges of parameter selections ensure consistent machining operations and consider the substantial effects that parameters have on roughness and MRR.

**Table 4:** Independent variables and levels.

Independent factor	Unit	Notation	Level		
			− 1	0	1
Nanoclay	%	A	1	3	5
Spindle speed	rpm	B	1500	2000	2500
Feed rate	mm/rev	C	200	300	400
Depth of cut	mm	D	0.5	1	1.5

In the Box–Behnken design (BBD) approach, the levels of independent variables of the process variables are identified. Interaction of selected variables with corresponding responses was created using response surface methodology (RSM). Referring to previous studies, a 29 experiment design was used as a way of optimizing delamination in the drilling of plastic fiber-reinforced plastic composites using three process variables with levels identified using the Taguchi method. However, in this study, a total of 29 experiment designs were carried out, having four independent variables with levels of three, as shown in Table 4, where various levels of different variables are identified.

Milling was done on a vertical computer numerical control (CNC) milling machine with a maximum spindle speed of 4000 rpm supplied by HAAS Automation Inc., USA. The surface finish and surface roughness measurement were done using a Mitutoyo Talysurf surface finish tester. The surface quality and surface roughness of the machined composite samples with a sample size of 100 × 100 × 10 mm^3^ were examined. Table 5 presents the results from the milling experiment.

**Table 5:** Response results of trials based on Box–Behnken design.

Run	Factor 1	Factor 2	Factor 3	Factor 4	Response 1	Response 2
	A: Nanoclay (%)	B: Spindle speed (rpm)	C: Feed rate (mm/min)	D: Depth of cut (mm)	Surface roughness Ra (µm)	MRR (mm^3^/min)
1	3	1500	400	1	2.55	4000
2	3	2000	400	1.5	2.45	6000
3	1	1500	300	1	1.85	3000
4	3	2000	300	1	1.7	3000
5	1	2000	300	1.5	2.05	4500
6	3	2500	200	1	1.45	2000
7	3	2500	300	0.5	1.55	1500
8	5	2500	300	1	2.65	3000
9	3	2000	300	1	1.68	3000
10	3	1500	300	1.5	2.35	4500
11	3	1500	300	0.5	1.85	1500
12	5	2000	400	1	3.05	4000
13	3	2000	200	0.5	1.75	1000
14	3	2500	400	1	2.15	4000
15	3	1500	200	1	1.95	2000
16	1	2500	300	1	1.4	3000
17	3	2000	400	0.5	1.95	2000
18	5	1500	300	1	2.85	3000
19	1	2000	300	0.5	1.55	1500
20	5	2000	200	1	2.55	2000
21	1	2000	400	1	1.95	4000
22	3	2000	300	1	1.69	3000
23	3	2000	200	1.5	1.95	3000
24	5	2000	300	1.5	3.15	4500
25	5	2000	300	0.5	2.35	1500
26	3	2500	300	1.5	1.75	4500
27	3	2000	300	1	1.67	3000
28	3	2000	300	1	1.66	3000
29	1	2000	200	1	1.55	2000

In conducting analyses of variance (ANOVA), both analyses were carried out using coded levels of factors and type III sum of squares. This was because using coded factors makes it possible to compare contributions of process factors, and type III sum of squares analyses take into consideration all other terms to provide a suitable response surface analysis technique for multiple factors.

The model F-value of 41.08 shows that the developed regression model is statistically significant with a very low probability of occurrence due to random error (*p* < 0.0001) (Table 6). This serves as a strong justification for saying that the regression model can be used for the analysis of machining properties for nanoclay-filled GRP composites.

**Table 6:** ANOVA analysis of surface roughness.

Source	Sum of squares	df	Mean square	F-value	*p* value	
Model	6.42	14	0.4583	41.08	< 0.0001	Significant
A-Nanoclay	3.26	1	3.26	291.74	< 0.0001	
B-Spindle speed	0.5002	1	0.5002	44.83	< 0.0001	
C-Feed rate	0.7008	1	0.7008	62.81	< 0.0001	
D-Depth of cut	0.6075	1	0.6075	54.45	< 0.0001	
AB	0.0156	1	0.0156	1.40	0.2564	
AC	0.0025	1	0.0025	0.2241	0.6433	
AD	0.0225	1	0.0225	2.02	0.1775	
BC	0.0025	1	0.0025	0.2241	0.6433	
BD	0.0225	1	0.0225	2.02	0.1775	
CD	0.0225	1	0.0225	2.02	0.1775	
A^2^	1.14	1	1.14	101.74	< 0.0001	
B^2^	0.0565	1	0.0565	5.06	0.0410	
C^2^	0.2918	1	0.2918	26.15	0.0002	
D^2^	0.1219	1	0.1219	10.92	0.0052	
Residual	0.1562	14	0.0112			
Lack of fit	0.1552	10	0.0155	62.08	0.0006	Significant
Pure error	0.0010	4	0.0003			
Cor total	6.57	28				

*P* values below 0.0500 show statistically significant terms in the models. In the current investigation, the linear terms nanoclay content (A), spindle speed (B), feed rate (C), and depth of cut (D), along with their counterparts A^2^, B^2^, C^2^, and D^2^, are found to be statistically significant. This verifies the significant individual effects of various machining variables on surface roughness and the significant effects of strong non-linearities in the chosen region of experiments. On the other hand, terms in models having *p* values greater than 0.1000 are found to be statistically insignificant, which anticipates the absence of significant effects of these terms on surface roughness. A significant number of statistically insignificant interaction terms also justify considering model reduction, considering hierarchy, to increase the simplicity of models.

The F-value of the lack-of-fit is 62.08 with a corresponding probability of 0.06%, which means that the lack of fit is statistically significant. Generally, the statistically significant lack of fit gives evidence that the model does not capture all the variability contained in the experimental data. This may be attributed to the inherent complexity and heterogeneity of nanoclay-filled GRP composites, local tool–workpiece interaction phenomena, and other unmodeled factors such as tool wear progression, machine vibration, and thermal effects during machining. The statistically significant lack of fit notwithstanding, the reasonably small value of pure error confirms a good repeatability of the experiment. Besides, a high value of model F and close agreement between predicted and experimental responses imply that the developed model remains efficient and reliable within the studied range of machining parameters for process optimization.

### Model adequacy and diagnostic analysis

**Fig. 3:**
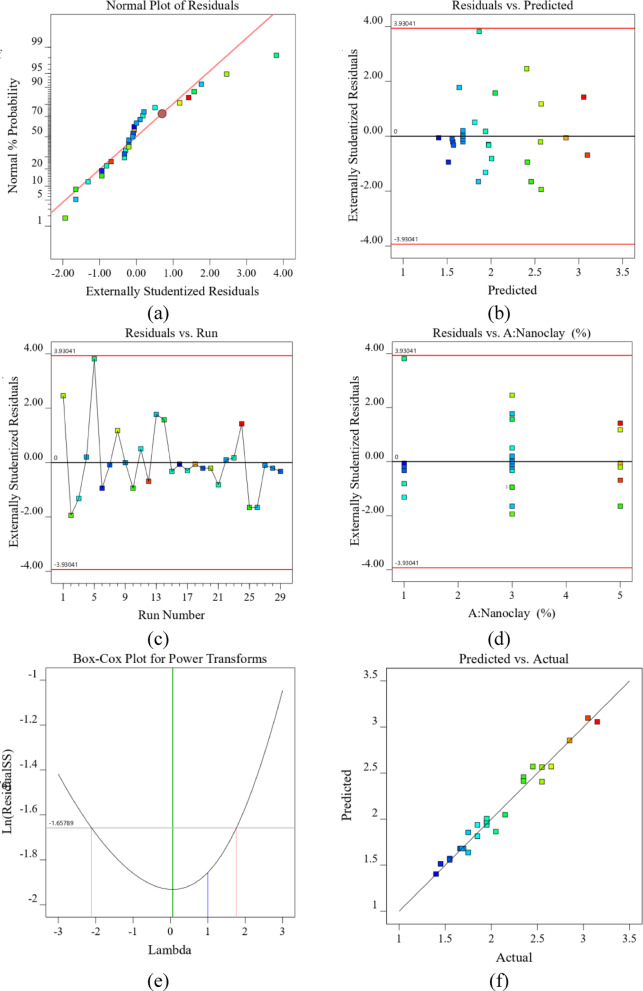

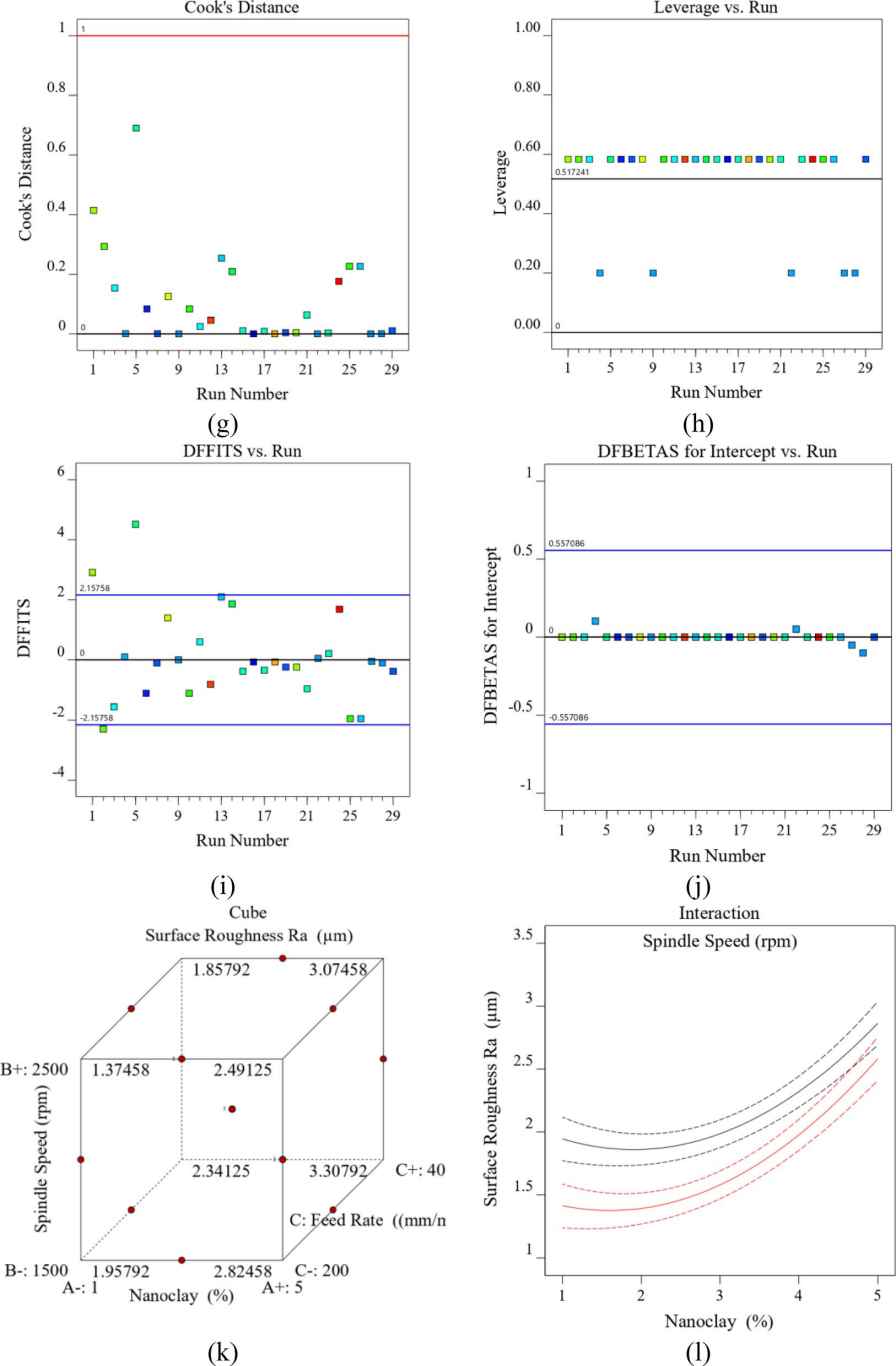

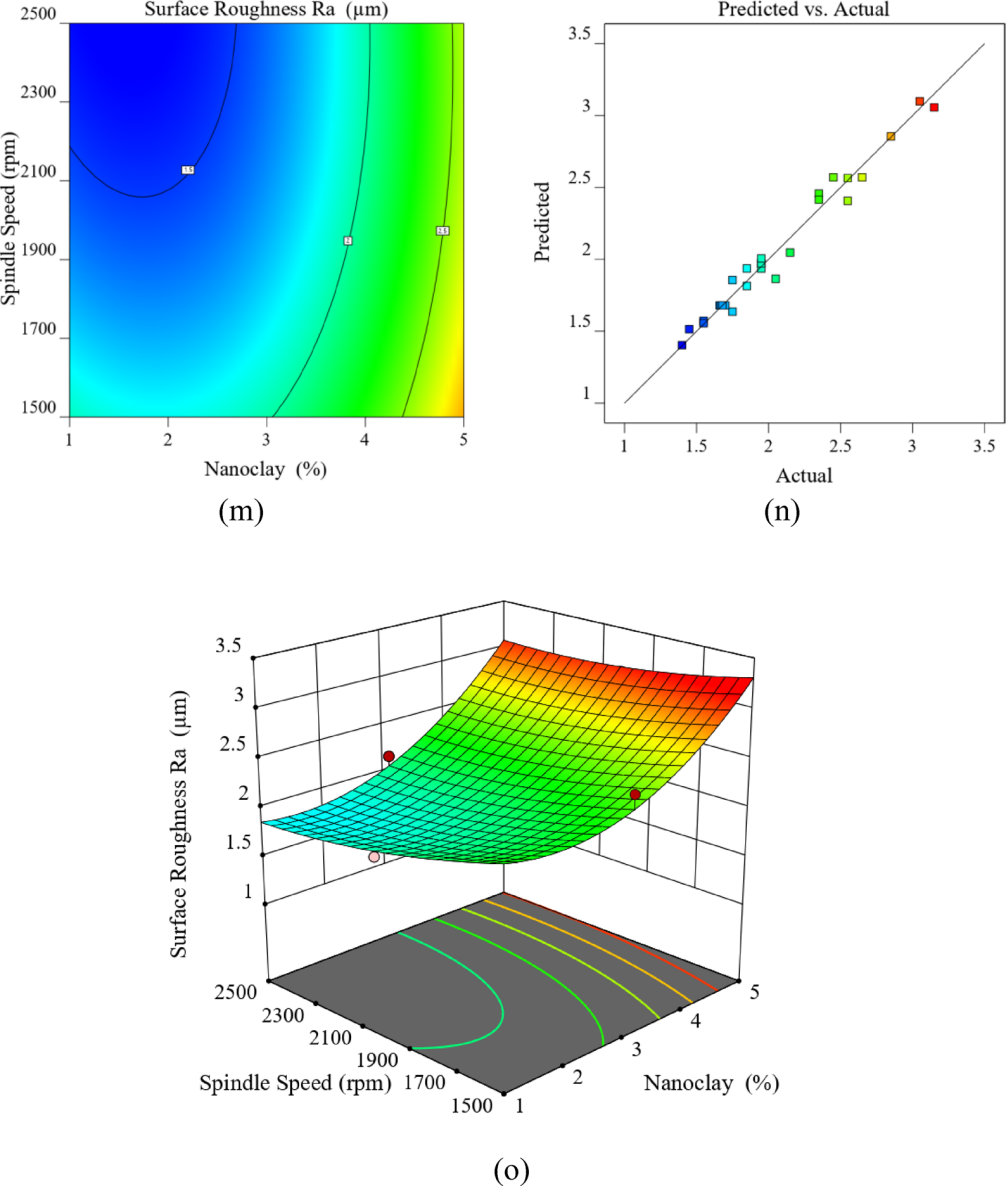
**a**–**o** Response Surface Methodology (RSM) model for predicting surface roughness (Ra) during CNC milling machining of nanoclay-filled GRP composites.

The appropriateness of the developed response surface methodology (RSM) model in predicting the surface roughness (Ra) in CNC machining of nanoclay-filled GRP composites was verified through a set of graphical diagnostic tools shown in Fig. 3a–o. From the normal probability plot of the residuals (Fig. 3a), it is found that the residuals plot closely to a straight line. This implies the normality of the residuals. This ensures that the regression analysis satisfies the normality assumption. There is no skewness and/or kurtosis in the graph; that is, there is a good fit between the observed and predicted values. The graph of predicted vs. actual (Fig. 3e,n) again confirms the correctness of the generated model. The points of actual value tend to be close to the 45° line, proving the closeness of the actual value to the predicted value of surface roughness. This again proves the dependability of the RSM on this surface roughness value range. In the plot of residuals vs. predicted values (Fig. 3j), there is random scattering of residuals around the line of zero residuals, and there are no patterns or funnels. Thus, there is constant variance or homoscedasticity, which satisfies the requirements of the regression assumptions. Also, from Fig. 3k, residuals vs. run order, there are no patterns, which ensures the absence of errors based on time or experiment due to machining. This scatter diagram of residuals and nanoclay percentage (Fig. 3) shows equal dispersion of data points for varying nanoclay Percentage, which means that the contribution of nanoclay Percentage has been appropriately accounted for in this model and does not require transformation of the response.

### Influence diagnostics and outlier detection

Influence diagnostics: Influence diagnostics were evaluated based on Cook’s distance, DFBETAS, DFFITS, and leverage plots (Fig. 3b,g,i,m). All simulation runs fall within the threshold of acceptability, and this indicates that there is no single data point with excessive influence on regression coefficients and predicted values. This verifies the statistical integrity of the Box–Behnken experimental design. In the leverage versus run graph, it can be seen that all points lie within the bounds of the warning zone (Fig. 3m). This helps to ensure that a safe and efficiently distributed space has been searched by the experiment. The Box-Cox plot (Fig. 3o) shows that the value of lambda (λ) is close to 1 and falls within the 95% confidence interval. Thus, there is no need to transform the data on surface roughness, and the original data can be used in regression studies.

### Interaction and surface response analysis

Machining parameters interface effects on surface roughness are presented using interaction plots, contour plots, and three-dimensional surface response plots (Fig. 3c,d,f,h). The interaction plot for spindle speed and nanoclay (Fig. 3c) shows that there is a significant interaction effect. At lower spindle speed, the surface roughness values reduce with the addition of nanoclay because of enhanced interfacial bonding and matrix-reinforcement load transfer. Conversely, at higher spindle speeds, the surface values increase because of the excessive heat generated during the machining, softening, and fiber pull-out. The contour plot (Fig. 3d) indicates an optimal region that corresponds to a certain amount of nanoclay and spindle speed. The optimal region is where the lowest value of the surface roughness is obtained. After the optimal amount of nanoclay, the value of the surface roughness increases due to agglomeration. A three-dimensional response surface graph (Fig. 3f) clearly shows that at the initial stages of nanoclay addition, the surface roughness decreases; however, afterwards it again increases. It can be denoted that nanoclay aggregation takes place in the nanocomposite.

The cube factor graph (Fig. 3h) represents the interaction between nanoclay content, spindle speed, and feed rate. Lower surface roughness values are noticed in the vicinity of the optimised point with moderate nanoclay content, low feed rate, and medium spindle speed value. This reveals the significance of multi-variable optimization in CNC machining of nanocomposites (Table 7).

**Table 7:** ANOVA analysis MRR.

Source	Sum of squares	df	Mean square	F-value	*p* value	
Model	3443.32	14	245.95	9116.18	< 0.0001	Significant
A-Nanoclay	0.0000	1	0.0000	0.0000	1.0000	
B-Spindle speed	0.0000	1	0.0000	0.0000	1.0000	
C-Feed rate	1006.19	1	1006.19	37,294.20	< 0.0001	
D-Depth of cut	2388.45	1	2388.45	88,527.58	< 0.0001	
AB	0.0000	1	0.0000	0.0000	1.0000	
AC	0.0000	1	0.0000	0.0000	1.0000	
AD	0.0000	1	0.0000	0.0000	1.0000	
BC	0.0000	1	0.0000	0.0000	1.0000	
BD	0.0000	1	0.0000	0.0000	1.0000	
CD	22.99	1	22.99	851.99	< 0.0001	
A^2^	0.0001	1	0.0001	0.0048	0.9456	
B^2^	0.0001	1	0.0001	0.0048	0.9456	
C^2^	3.94	1	3.94	146.21	< 0.0001	
D^2^	22.38	1	22.38	829.40	< 0.0001	
Residual	0.3777	14	0.0270			
Lack of fit	0.3777	10	0.0378			
Pure error	0.0000	4	0.0000			
Cor total	3443.70	28				

The analysis of variance (ANOVA) is performed in the coded form of the factors, and the Type III (Partial) sum of squares is used to assess the individual effect of each term in the models, taking into consideration the effect of all other factors. The Type III sum of squares is most useful when there is more than one variable in the response surface models.

The remarkably high value of Model F, which is 9116.18, proves that the regression model built is significantly accurate. The probability related to the statistic shows that there is only a 0.01% chance for such a high value to occur due to randomness. Such accuracy confirms the capability of this regression model to represent the relationship for the chosen parameters with a high level of accuracy. *p* values < 0.0500 indicate significant model terms. The significant model terms for this research problem are the linear terms C and D, the interaction term CD, and the quadratic terms C^2^ and D^2^. This implies that the process is dominantly affected by these terms, and their linear as well as nonlinear impact is a significant factor. The significant effect of the interaction term CD implies that the simultaneous effect of the terms C and D has a significant effect on the process.

On the other hand, those terms in the model that have *p* values in excess of 0.1000 are considered statistically insignificant; that is, they do not affect the variation of the response. It appears that there are a few insignificant terms in the model, which suggests that a reduction in the model, while preserving hierarchy in the new model, could be useful.

**Fig. 4:**
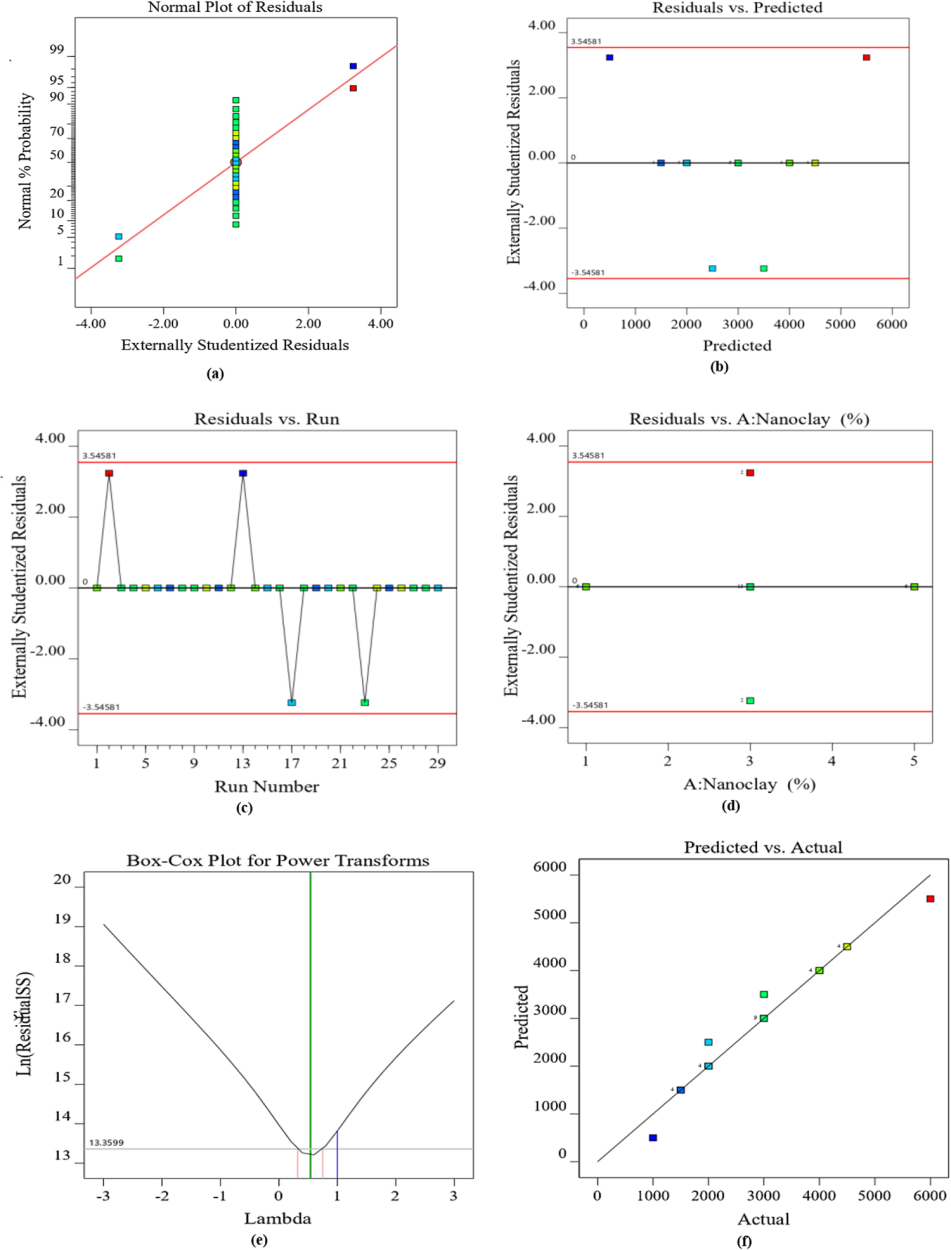

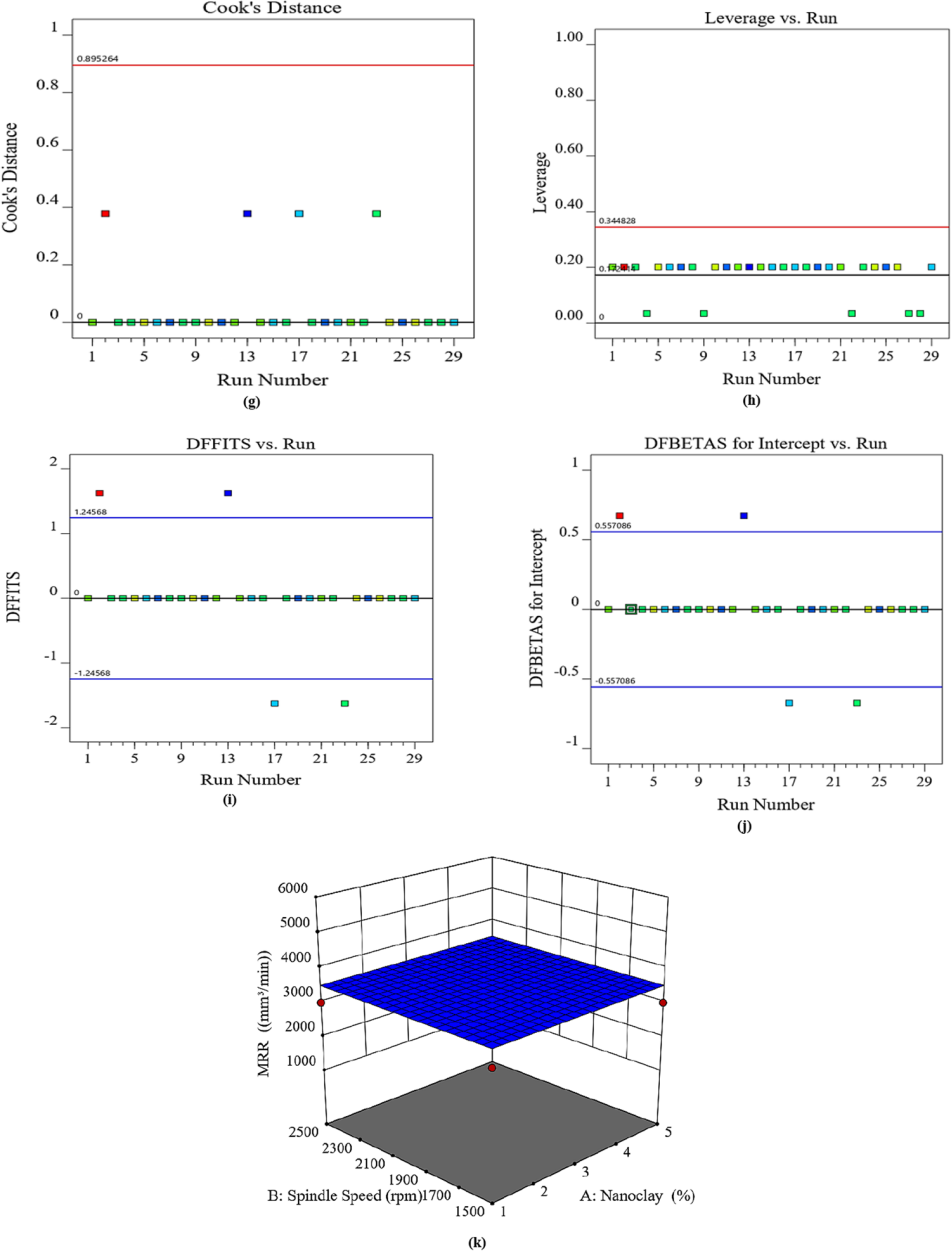
(**a**–**k**) Response surface methodology (RSM) model for predicting MRR during CNC milling machining of nanoclay-filled GRP composites.

Figure 4a–k shows the diagnostic and response surface graphs used to determine the adequacy and accuracy of the established response surface model. From the normal probability plot of residuals (Fig. 4a), the residuals appeared to follow a straight-line pattern, and this satisfies the normality assumption in the ANOVA test. The plot of the residuals and the predicted values (Fig. 4b) showed a scattered pattern around the zero line, and this indicated equal variance and lack of bias in the model. The plot of the predicted value versus the actual value (Fig. 4c) confirms the excellent level of consistency between the measured and predicted data. The plot of residuals against the order of the experiment (Fig. 4d) shows no trend. The interaction plot of factors C and D (Fig. 4e) indicates that there is a significant interaction effect, as seen from the ANOVA table. The contour plot of this function (Fig. 4f) helps to see the region of optimum for the response variable. The three-dimensional surface plot (Fig. 4g) further helps to understand the nature of the effect of these parameters on the response variable. The perturbation plot (Fig. 4h) suggests the dominant parameters for the response variable. The cook distance and leverage plots (Fig. 4i,j) confirm there are no influential data points. The Box and Cox plot (Fig. 4k) reveals there is no need for transformation of the responses. Thus, based on the graphical analysis, the adequacy and optimizing ability of the developed model are confirmed.

**Fig. 5:**
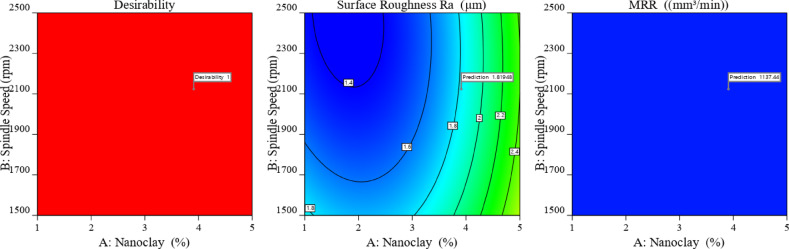
Contour plots for desirability-based optimization of Ra and MRR.

**Fig. 6:**
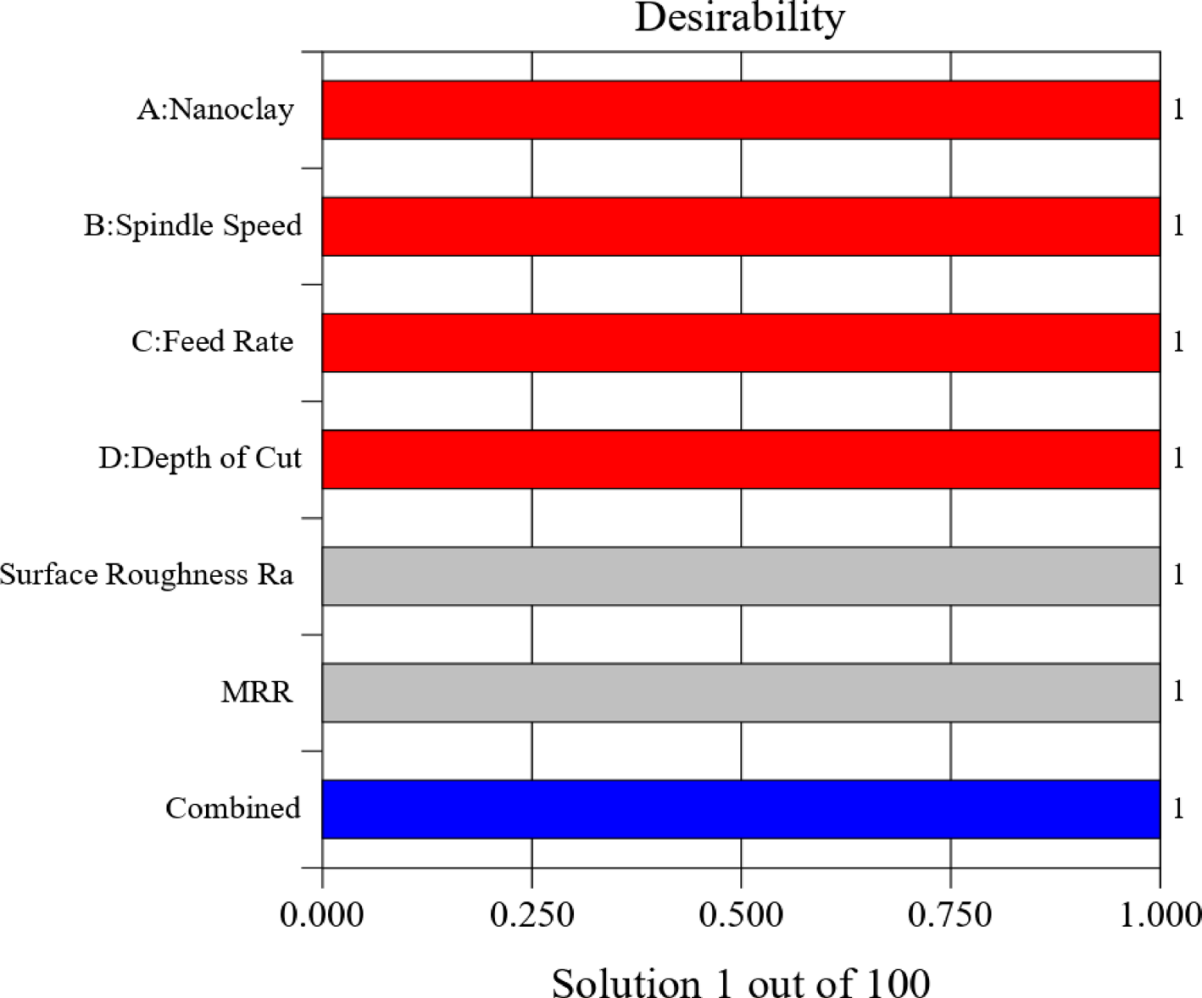
Desirability function analysis for multi-response optimization.

Figure 5 shows the contour plots developed from desirability-based multiresponse optimization studies, which give the combined effect of nanoclay content (A) and spindle speed (B) on desirability, surface roughness (Ra), and material removal rate (MRR). From the desirability plot, it can be observed that the value of desirability is uniformly high (close to unity) throughout the selected design space. This confirms that the combination of process parameters chosen by the optimization routine satisfies the optimization objectives simultaneously. The highest value of desirability corresponds to a nanoclay content of about 3.9 wt% and a spindle speed of about 2123 rpm. This confirms the presence of an optimal machining regime where surface quality and productivity are balanced out effectively. The contour plot of surface roughness indicates a strong dependence of Ra on nanoclay and spindle speed. The lowest surface roughness values occur for moderate nanoclay concentrations and moderate spindle speeds. At higher concentrations of nanoclay, a rise in surface roughness occurs due to agglomeration of particles and an increased interaction between the tool and matrix, which produces deleterious effects on surface finish. At higher spindle speeds, an increase in surface roughness also occurs because of material softening, vibrations, and fiber pull-out. Minimum surface roughness, as estimated in the optimum zone, occurs around 1.82 µm. The MRR contour plot clearly illustrates the stable and high MRR obtained for the machining process over the explored ranges of nanoclay concentration and spindle speed. The calculated MRR at the optimum point is found to be approximately 1137 mm^3^/min. This clearly illustrates that high MRR can certainly be obtained without compromising the surface finish of the machined workpiece. The lower variability for MRR implies that the effect of nanoclay concentration is insignificant for material removal rate. As Fig. 6 indicates, the desirability plot shows that all the selected machining parameters (nanoclay content, spindle speed, feed rate, and depth of cut) attain a desirability value of unity under optimized conditions. Both response variables, that is, surface roughness, Ra, and material removal rate, MRR, correspond to their respective desirability goals while yielding a combined desirability of unity. It concludes that the investigated parameter combination corresponds to an optimal solution, which successfully leads to satisfaction of the multi-objective optimization criteria by minimizing the surface roughness and maximizing MRR under the combined effect of both objectives.

## Conclusion

The findings provide empirical evidence that controlled addition of nanoclay reinforcement improves both mechanical and machinability properties in GFRP composites. These properties include the following quantitative outcomes:

Density: An increasing trend in specific gravity was measured from 1.836 (0%) nanoclay loading up to 1.858 (3%) nanoclay loading, resulting in 1.16% increase in matrix densification.

Tensile Properties: At 3% nanoclay, the composite showed the best tensile strength with a value of 283 MPa (18% increase from neat GFRP of 240 MPa) and tensile modulus of 23.1 GPa (11% increase from neat GFRP of 20.8 GPa). Further increase to 5% caused agglomeration effect and decreased the tensile strength by 11% to 252 MPa.

Flexural properties: Maximum flexural strength (25% increase from neat GFRP) and flexural modulus (27% increase from neat GFRP) were 525 MPa and 23.5 GPa, respectively. Peak load was 1180 N. The 5% nanoclay loading decreased flexural strength by 12% to 470 MPa and flexural modulus by 13% to 21.5 GPa due to the agglomeration effect.

Surface Roughness: The ANOVA-validated RSM model (F = 41.08, R^2^ > 0.95) confirms nanoclay content as the dominant factor influencing Ra (F = 291.74), followed by feed rate (F = 62.81), depth of cut (F = 54.45), and spindle speed (F = 44.83). Minimum surface roughness of ~ 1.82 μm was achieved at the optimal condition of ~ 3.9 wt% nanoclay and ~ 2123 rpm spindle speed.

MRR: The RSM model for MRR (F = 9116.18, *p* < 0.0001) confirms that depth of cut (F = 88,527.58) and feed rate (F = 37,294.20) are the dominant MRR drivers, while nanoclay content has a negligible influence (*p* = 1.000). Maximum MRR of ~ 1137 mm^3^/min was simultaneously achieved at the optimal condition.

Multi-response optimization using the desirability function yielded a perfect combined desirability of 1.000 (Solution 1 from 100 Runs), confirming that minimizing Ra and maximizing MRR simultaneously is achievable at ~ 3.9 wt% nanoclay, ~ 2123 rpm spindle speed, within the studied feed rate and depth of cut ranges. These optimized nanoclay-filled GFRP composites are recommended for aerospace structural panels, automotive body components, marine hull structures, and wind turbine blade applications where superior surface quality and machining efficiency are simultaneously critical.

## Data Availability

The datasets used and/or analysed during the current study available from the corresponding author on reasonable request.
